# Cactus cladodes associated with urea and sugarcane bagasse: an alternative to conserved feed in semi-arid regions

**DOI:** 10.1007/s11250-019-01895-1

**Published:** 2019-04-25

**Authors:** Thamires Damascena Quirino Siqueira, João Paulo Ismério dos Santos Monnerat, Juana Catarina Cariri Chagas, Maria Gabriela da Conceição, Michelle Christina Bernardo de Siqueira, Thays Bianca Lira Viana, Marcelo de Andrade Ferreira

**Affiliations:** 1 Department of Animal Science, Federal University of Pernambuco, Recife, PE 52171-900 Brazil; 2 NJV, Swedish University of Agricultural Sciences, 901 83 Umeå, SE Sweden

**Keywords:** Cactaceae, Non-protein nitrogen, Dry land, System production sustainability

## Abstract

The objective of this study was to evaluate the nutritional value of different roughage sources as an exclusive feed for goats and sheep from the determination of nutrient intake and digestibility. Five goats and five sheep were used and arranged in a double 5 × 5 Latin square design. Treatments consisted of cactus *Nopalea cochenillifera* (L.) Salm-Dyck cladodes (*Nopalea*) + urea + sugarcane bagasse (NUB), cactus *Opuntia stricta* (Haw.) Haw cladodes (*Opuntia*) + urea + sugarcane bagasse (OUB), Tifton hay (TH), maize silage (MS), and forage sorghum silage (SS). Dry matter (DM) and crude protein (CP) intakes of NUB were greater than SS (0.620 and 0.058 versus 0.416 and 0.040 kg/day). Metabolizable energy (ME) intake was higher for NUB (1.52 Mcal/day). The DM digestibility did not change between the roughages (59%). The NUB, OUB, and MS organic matter digestibility (OMD; 62.4%) were greater than that of SS (57.4%). The roughage consisting of cactus cladodes associated with urea and sugarcane bagasse presented a greater nutritional value, similar to MS and TH, and higher than SS. Cactus cladodes associated with urea and sugarcane bagasse are recommended as an alternative to conserved feed.

## Introduction

Forage availability, in terms of both quality and quantity, is the greatest impediment to animal production in semi-arid regions; this is mainly due to irregular rainfall. The use of conserved fodder, such as silages and hay, is common to ensure the feeding of livestock throughout the year; however, given the edaphoclimatic conditions, the production of conserved forage is still limited.

In face of the adversity of the arid and semi-arid regions, cactus cladodes are widely used around the world (Oliveira et al. 2018); they present high green matter production, even in hydric stress (Ben Salem 2010), and are greatly palatable to ruminant animals (Leite et al. 2014). The Brazilian semi-arid region, which represents approximately 970,000 km^2^ (Brasil 2005), has been suffering from a period of drought since 2012 (Marengo et al. 2016). In the same time period, and associated with this drought, cactus cladode plantations were attacked by cochineal insect-pests (*Dactylopius coccus*), practically depleting all forage stocks.

Given the high dependence on livestock in the semi-arid region, producers needed to acquire external inputs, resulting in the commercial production of roughage in northeastern Brazil and irrigated areas to produce maize and forage sorghum silage, Tifton hay, and sugar cane sold at high prices. This activity solved the problem in the short-term; however, in the medium- and long-term, it could impair the sustainability of the local livestock production.

Thus, the restoration of the cactus cladode field became necessary. In this region, two resistant cultivars for ruminants have been studied: cactus *Nopalea cochenillifera* (L.) Salm-Dyck cladodes and cactus *Opuntia stricta* (Haw.) Haw. cladodes. Although cactus cladodes are an energy- and water-rich feedstuff, they have low protein and fiber contents, restricting their inclusion in ruminant diets. Nonetheless, these deficits may be balanced with the inclusion of a non-protein nitrogen source, such as urea and roughage that is high in physically effective neutral detergent fiber (NDF). In this context, the utilization of agro-industrial residues that are commonly rich in fiber, such as oat, maize, and wheat straw, as well as sugarcane bagasse, can be used to balance the low NDF content of cactus cladodes-based diets (Almeida et al. 2018).

The sugarcane bagasse, a result of juice extraction after crushing in the mills, is the largest residue of Brazilian agroindustry. For each cane ground ton, 700 l of cane juice and 300 kg of bagasse (50% of dry matter) are obtained. The bagasse was all used as fuel in the industry’s own boilers, replacing wood. And more than 15 years ago, different industrial sectors began to use the bagasse as a fuel, making the mills and distilleries invest to optimizing the boilers and turbines, increasing the bagasse production. Nowadays, the sugarcane bagasse excedent is about 20% of the total production (Teixira et al. 2007).

It was hypothesized that the combination of cactus *Nopalea* and cactus *Opuntia* cacti with sugarcane bagasse, a waste residue of the sugar and alcohol industry, as well as urea, may present a similar nutritional value as traditional roughages, such as maize and forage sorghum silages, as well as Tifton hay. Therefore, this study aimed to evaluate the nutritional value of different roughage sources for sheep and goats from the determination of their nutrient intake and digestibility.

## Materials and methods

This experiment was conducted at the Laboratory of Feed Evaluation for Small Ruminants II of the Animal Science Department of the Federal Rural University of Pernambuco (UFRPE), Recife, Brazil.

Five female Saanen goats and five female crossbred sheep (*Somalia Brasileira x Morada Nova x Bergamácia Brasileira*), at body weight of 27.61 kg (± 4.35 kg) and age of 6 months, were distributed in a double 5 × 5 Latin square design. The experimental animals were selected based on sex, age, and initial body weight. The animals were housed in metabolism cages and provided with a feeder and drinkers. The trial lasted 105 days, with five consecutive 21-day periods, which were divided into 14-day adaptation and 7-day sampling periods.

The five experimental diets were composed of different roughages; additionally, common salt and vitamin-mineral premix were provided (Tables 1 and 2). Treatments consisted of cactus *Nopalea cochenillifera* (L.) Salm-Dyck cladodes (Nopalea) + urea:AS + sugarcane bagasse (NUB), cactus *Opuntia stricta* (Haw.) Haw cladodes (Opuntia) + urea:AS + sugarcane bagasse (OUB), Tifton hay (TH), maize silage (MS), and forage sorghum silage forage (SS). The cactus cladodes were offered chopped and mixed with the other diets ingredients. Tifton hay (10.1% crude protein) was adopted as a protein source. The maize silage (Agroceres® AG5055 cultivar: yellow-orange and early-cycle medium grain) and sorghum silage (SF-15) had the percentage of crude protein (CP) corrected with urea:AS (9:1). For roughages based on cactus cladodes, sugarcane bagasse was used for NDF correction and urea was used to ensure adequate protein. Feeding was offered ad libitum as a mixed ration at 08 h00 and 16 h00, and orts were weighed daily before the morning feeding in order to estimate the animals’ intake.

**Table 1:** Chemical composition of ingredients used in the experimental diets (DM, %)

Item	Roughage					
	*Nopalea* ^1^	*Opuntia* ^2^	SB^3^	TH^4^	MS^5^	SS^6^
DM	11.1	11.9	89.8	85.8	25.2	23.3
OM	86.8	89.00	93.5	91.4	94.1	91.8
CP	3.40	4.00	1.1	10.1	8.9	6.00
NDIP	1.27	1.06	0.34	1.43	0.73	0.62
NDFap	26.0	30.2	85.9	74.7	61.9	69.4
iNDF	9.7	11.9	45.6	29.6	18.1	22.9
ADF	17.2	17.8	64.2	44.8	42.6	46.9
NFC	64.5	56.3	5.5	5.9	19.7	12.8

^1^Cactus *Nopalea cochenillifera* (L.) Salm-Dyck cladodes; ^2^Cactus *Opuntia stricta* (Haw.) Haw. cladodes; ^3^Sugarcane bagasse; ^4^Tifton hay; ^5^Maize silage; ^6^Forage Sorghum silage

**Table 2:** Proportion of ingredients and chemical composition of the roughages

Ingredient, %	Roughages				
	^1^NUB	^2^OUB	^3^TH	^4^MS	^5^SS
Maize silage	–	–	–	97.89	–
Sorghum silage	–	–	–	–	96.62
Tifton hay	–	–	97.99	–	–
Cactus *Nopalea*	61.54	–	–	–	–
Cactus *Opuntia*	–	60.44	–	–	–
Sugarcane bagasse	33.62	34.80	–	–	–
Urea/AS^6^	2.76	2.61	–	0.26	1.50
Mineral mixture	2.08	2.16	2.01	1.75	1.88
Nutritional composition, %					
DM	16.76	18.13	86.03	25.60	23.92
OM	84.85	86.33	89.56	91.11	88.70
CP	9.82	9.70	9.90	9.40	9.78
NDF	44.88	48.15	73.20	60.59	67.06
iNDF	21.30	23.10	29.00	17.70	22.20
NFC	36.50	34.30	5.90	21.40	14.20
ME (Mcal/kg of DM)	2.43	2.51	2.23	2.26	2.10

^1^Cactus *Nopalea* + urea + sugarcane bagasse; ^2^Cactus *Opuntia* + urea + sugarcane bagasse; ^3^Tifton hay; ^4^Maize silage; ^5^Forage Sorghum silage; ^6^Urea + ammonium sulfate (9:1)

Voluntary intake was evaluated from the 15th to 21st day of the experimental period; in this sense, the amounts of supplied feed and orts were considered. In the digestibility trial, a total collection of faeces was taken on the 16th, 17th, and 18th days of each experimental period (72 h), starting and finishing at 06 h00. Faeces were collected through the metabolism cage. At the end of each collection day, faeces were weighed and homogenized, and an aliquot of approximately 50 g/kg was taken and frozen at − 18 °C, as well as samples of orts and feed that were taken over the same trial period.

Feeds, orts, and faeces samples were dried in a forced ventilation oven (60 °C) for 72 h and processed in a Willey mill with 1- and 2-mm sieves. Subsequently, samples were compiled per animal and experimental period. The samples that were processed to pass through the 1-mm screen sieve were evaluated for dry matter (DM; method INCT-CA G-003/1), organic matter (OM; method INCT-CA M-001/1), CP (method INCTCA N-001/1), ether extract (EE; method INCT-CA G-005/1), neutral detergent fiber corrected for ash and protein (NDFap; methods INCT-CA F-002/1, INCT-CA M-002/1, and INCT-CA N-004/1), acid detergent fiber (ADF; methods INCT-CA F-004/1), and neutral detergent insoluble protein (NDIP; method INCT-CA N-004/1) according to the standard techniques of the Brazilian National Institute of Science and Technology in Animal Science (INCT-CA; Detmann et al. 2012). Feeds and orts samples that were processed to pass through the 2-mm screen sieve were evaluated for indigestible NDF (iNDF; method INCT- CA 009/1) content using a 288-h in situ incubation procedure. The quantification of non-fiber carbohydrates (NFC) was performed according to Detmann and Valadares Filho (2010) as follows: NFC = OM − [(% CP − % CP of urea + % urea:AS) + % NDF + % EE + MM]. The total digestible nutrient (TDN) content of the diets was determined according to Weiss et al. (1992), and its conversion to metabolizable energy (ME) was estimated by Detmann et al. (2016).

The DM prices per kg adopted for the final estimated costs of each roughage were urea:AS (US$ 0.68), salt (US$ 0.16), minerals (US$ 0.81), silages (US$ 0.47), and hay (US$ 0.49); these were the current prices in the livestock market. The cactus cladode prices were taken from Donato et al. (2017), corrected to the year 2019. The dollar conversion rate was US$ 1.00 = R$ 3.10.

Relative data on intake and total digestibility were analyzed using the MIXED procedure in SAS (version 9.4), according to the following model:

$$ \mathrm{Yijk}=\mu +\mathrm{Ti}+\mathrm{Qj}+\mathrm{Pk}+\left(A/Q\right) lj+{T}^{\ast}\mathrm{Qij}+\upvarepsilon \mathrm{ijk} $$

where Yijk = observation ijk; *μ* = over mean; Ti = fixed effect of treatment *i*; Qj = fixed effect of square *j*; Pk = random effect of period *k*; (A/Q)lj = random effect of animal *l* into square *j*; T*Qij = fixed effect of treatment *i* and square *j* interaction; εijk = random residual error.

The means were compared with a Tukey’s test. A significance value of 0.05 was adopted as the critical value of the probability of type I error.

## Results

Relating to the different roughages, there was almost no difference in the chemical composition between the cactus *Nopalea* and cactus *Opuntia*, and both were near the values presented in the Brazilian Tables (Cqbal; Valadares Filho et al. 2018). Due to the similar chemical composition observed for roughages based on cactus cladodes (Table 3), the results found were also similar in between them. The conserved roughage (MS, SS, and TH) used in the semi-arid region and adopted in this trial presented different chemical composition values for DM, NDF, ADF, and NFC, as verified by Valadares Filho et al. (2018; Table 3).

**Table 3:** Chemical composition of Tifton hay, maize silage, and forage sorghum silage

Item, %	Tifton hay		Maize silage		Forage Sorghum silage	
	Actual	Cqbal^1^	Actual	Cqbal^1^	Actual	Cqbal^1^
DM	85.8	88.7	25.2	31.1	23.3	28.1
OM	91.4	92.4	94.1	94.3	91.8	93.6
CP	10.1	9.3	8.9	7.18	6.0	6.3
NDFap	74.7	71.9	61.9	50.2	69.4	56.3
iNDF	29.6	30.5	18.1	17.9	22.9	22.6
ADF	44.8	39.5	42.6	29.5	46.9	32.5
NFC	5.9	8.9	19.7	34.9	12.8	29.5

^1^Mean values according to Brazilian Tables (Cqbal; Valadares Filho et al. 2018)

There was no variation in the intake and digestibility of nutrients between the species (sheep and goats) in this study, and the interaction between roughage and species was not significant (*P* > 0.05). The intakes of DM (0.62 versus 0.42 kg/day) and CP (0.06 versus 0.04 kg/day) were higher for NUB than SS, respectively (Table 4). The OM intakes for NUB (0.52 kg/day) and MS (0.54 kg/day) were greater than SS (0.38 kg/day). The NDF intakes for TH (0.37 kg/day) and MS (0.35 kg/day) were greater than NUB (0.25 kg/day) and OUB (0.22 kg/day). The intakes of NFC for NUB (0.29 kg/day) and OUB (0.23 kg/day) were greater than MS (0.11 kg/day), which was higher than TH (0.03 kg/day). The ME intake for NUB (1.52 Mcal/day) was greater than TH (1.04 Mcal/day) and SS (0.89 Mcal/day).

**Table 4:** Nutrient intake and digestibility for sheep and goats fed different roughages

	Roughages					SEM	*P* value		
Item	NUB^1^	OUB^2^	TH^3^	MS^4^	SS^5^		Roughages	Species	R x S^6^
	Intake, kg/day								
DM	0.62 a	0.52 ab	0.50 ab	0.58 a	0.42 b	0.041	0.017	0.932	0.089
OM	0.52 a	0.45 ab	0.45 ab	0.54 a	0.37 b	0.037	0.023	0.943	0.112
CP	0.06	0.05	0.05	0.05	0.04	0.074	0.043	0.273	0.064
NDF	0.25 b	0.22 b	0.37 a	0.35 a	0.28 ab	0.024	< 0.001	0.634	0.096
NFC	0.29 a	0.23 a	0.03 c	0.11 b	0.05 bc	0.002	< 0.001	0.290	0.359
ME, Mcal/day	1.52 a	1.21 ab	1.04 bc	1.28 ab	0.89 c	0.026	< 0.001	0.933	0.092
Digestibility, %									
DM	58.03	59.33	58.67	62.30	56.80	2.464	0.601	0.171	0.566
OM	63.70 a	61.67 a	61.26 ab	61.80 a	57.36 b	0.971	0.002	0.184	0.653
CP	71.04 a	67.69 a	67.75 a	59.02 b	63.24 b	1.061	< 0.0001	0.653	0.633
NDF	52.29 c	48.89 c	63.07 a	58.47 b	60.43 ab	0.873	< 0.0001	0.772	0.871

^1^Cactus *Nopalea* + urea + sugarcane bagasse; ^2^Cactus *Opuntia* + urea + sugarcane bagasse; ^3^Tifton hay; ^4^Maize silage; ^5^Forage Sorghum silage; ^6^Roughages and animal species interaction. Means followed by different letters on the same line differ by the Tukey test (P < 0.05)

Regarding DM digestibility, there was no variation among the different roughages (*P* > 0.05; Table 4). The OM digestibilities of NUB (63.7%), OUB (61.67%), and MS (61.80%) were higher than SS (57.36%). The CP digestibilities of NUB (71.04%), OUB (67.69%), and TH (67.75%) were higher than MS (59.02%) and SS (63.24%). The NDF digestibility for TH (63.07%) was higher than MS (58.47%), which in turn, was higher than NUB (52.29%) and OUB (48.49%).

## Discussion

The tested silages presented lower dry matter and NFC contents and a greater cell wall content (NDF and ADF) than the values verified by Valadares Filho et al. (2018), especially the forage sorghum silage. Considering these differences and the current market prices, it can be inferred that, if livestock continue to depend on commercial roughages, the sustainability of the ruminant production system will be threatened.

The results for intake and digestibility were discussed jointly for both animal species since there was neither a species effect nor a roughages x species interaction. Nutrient intake is influenced by numerous factors, particularly nutrient content, the associative effects of dietary components, and feed processing (Macedo Júnior et al. 2009). The greater observed intake for NUB may be attributed to the high NFC content of this roughage (41.54%; Table 2). In addition, the roughage composed of cactus *Nopalea* may have been distinguished by its high palatability, corroborating with the results of a dairy cow study (Silva et al. 2018). The low observed intake for SS may also be associated with a high concentration of ADF (46.9%), which is associated with low nutrient digestibility, providing ruminal repletion, reducing the rate of passage and, thus, reflecting the low dry matter intake (Yang et al. 2001).

The OM intake followed the pattern of the DM intake, whereas the NFC intake was a function of the chemical composition of the different roughages (Table 2). The highest NFC proportion was observed for the roughage composed of cactus cladodes, presenting a higher NFC intake (0.262 kg/day) as a consequence, whereas the roughage with a lower proportion of NFC was TH (Table 3), presenting, consequently, a lower intake for this nutrient (0.026 kg/day).

The observed response in NDF intake was also associated with the chemical composition of the roughages (Table 1). Since the NDF contents were lower for the cactus cladode roughages (NUB and OUB), the NDF intake was also lower with these roughages (0.227 kg/day). The opposite pattern occurred for MS and TH, which presented higher proportions of NDF in their composition, promoting a greater intake of this nutrient (0.349 kg/day), following the observed DM intake. Even with the high NDF content of SS (Table 1), the DM intake for this roughage was low, making its NDF intake proportionally low. Lastly, the ME intake for NUB was explained by the high NFC intake, as well as the high digestibility of this nutrient (83%).

The increase in CP digestibility for NUB and OUB can be attributed to a higher non-protein nitrogen (NPN; 2.76 and 2.61% in the total diet) compared to the other roughages (Table 3), presenting a high ruminal disappearance rate of NPN as a function of its high solubilization (Jin et al. 2017).

The greater NDF digestibility observed for TH is related to the high concentration of structural carbohydrates in this roughage. These components present slower ruminal digestion rates when compared to the soluble carbohydrates present in the spineless cactus, which are rapidly degraded. Therefore, the higher retention time of hay in the rumen is associated with the higher fiber degradability provided by the extensive activity of the microorganisms on the feed (Siqueira et al. 2018).

The low NDF digestibility for the roughage composed of cactus cladodes, urea, and sugarcane bagasse (50.59%; Table 3) was probably due to the low digestibility of sugarcane bagasse (Gomes et al. 2012). Bagasse contributed to 65% of the total NDF of the NUB and OUB roughages. The low digestibility of the bagasse fiber, as well as the high concentration of the complex chemical bonds of lignin with cellulose and hemicellulose, is a topic that is widely discussed by researchers; this justifies the chemical treatment of this feed with calcium, for instance, which seeks to promote improvements in its nutritional value (Pimentel et al. 2015). The bagasse treated with calcium oxide reduces the NDF content and increase the digestibility; however, the presented fiber composition of the bagasse is what we aim to correct the cactus cladodes NDF content.

Considering the results of this study, and the limitations of forage production in semi-arid regions with low rainfall regularity, the importance of cactus cladodes for production systems in arid and semi-arid regions is evident. The roughages with the tested cactus cladodes were simple mixtures at a ratio of 62:3:35 (cactus cladodes:urea/AS:bagasse), making it possible to provide a similar energy level as other traditional conserved roughages (Tifton hay and maize silage) and a higher energy level than sorghum forage silage. This analysis becomes relevant when the DM costs per kg and the energy unit of each roughage can be compared. The roughages composed of cactus cladodes presented a much cheaper DM cost per kg compared to the other evaluated roughages, with the cactus cladodes promoting a lower cost per unit of energy (Table 5).

**Table 5:** Price per kg of DM and Mcal produced according to the roughages

Item	Roughages				
	NUB^1^	OUB^2^	TH^3^	MS^4^	SS^5^
ME (Mcal/kg of DM)	2.43	2.51	2.23	2.26	2.10
R$/kg of DM^6^	0.60	0.60	1.52	1.22	1.14
R$/Mcal^6^	0.25	0.24	0.68	0.54	0.58

^1^Cactus *Nopalea* + urea + sugarcane bagasse; ^2^Cactus *Opuntia* + urea + sugarcane bagasse; ^3^Tifton hay; ^4^Maize silage; ^5^ Forage Sorghum silage*;*^6^Considering the market prices for silages and hay; ^7^

According to this approach, for ruminant production in semi-arid regions, the necessity of cactus cladodes is becoming re-established; there is an increase in the demand for feed and a high risk of using conventional forages for fencing or silage production. Aiming to not depend on exterior inputs to maintain animal herds with similar available energy, we recommend the use of cactus cladodes associated with urea and sugarcane bagasse, which will guarantee lower diet costs and the sustainability of the production system.

In a performance trial with finishing lambs fed cactus cladodes diets (35% of total DM) associated with MS, TH, or SB, no difference were observed in the DMI 1.1 kg/day and average daily gain 0.19 kg/day (Ribeiro et al. 2017), which indicates that the roughages with cactus cladodes used in our experiment can be applicable to finishing lambs feeding. Thus, we have the technology package (bagasse+cactus+urea/AS) for a lower price than other options and known energetic value ready to formulate diets at different levels of nutrition requirement.
